# Effects of zinc in podocytes and cortical collecting duct *in vitro* and Dahl salt-sensitive rats *in vivo*

**DOI:** 10.1016/j.jbc.2024.107781

**Published:** 2024-09-12

**Authors:** Ruslan Bohovyk, Olha Kravtsova, Vladislav Levchenko, Christine A. Klemens, Oleg Palygin, Alexander Staruschenko

**Affiliations:** 1 Department of Molecular Pharmacology and Physiology, University of South Florida, Tampa, Florida, USA; 2 Hypertension and Kidney Research Center, University of South Florida, Tampa, Florida, USA; 3 Division of Nephrology, Department of Medicine, Medical University of South Carolina, Charleston, South Carolina, USA; 4 Department of Regenerative Medicine and Cell Biology, Medical University of South Carolina, Charleston, South Carolina, USA; 5 James A. Haley Veterans' Hospital, Tampa, Florida, USA

**Keywords:** zinc, podocyte, kidney, blood pressure, calcium

## Abstract

Zinc is one of the essential divalent cations in the human body and a fundamental microelement involved in the regulation of many cellular and subcellular functions. Experimental studies reported that zinc deficiency is associated with renal damage and could increase blood pressure. It was proposed that zinc dietary supplementation plays a renoprotective role. Our study aimed to investigate the effects of zinc on intracellular signaling in renal cells and explore the correlation between dietary zinc and the progression of salt-induced hypertension. The impact of extracellular zinc concentrations on two different kidney epithelial cell types, podocytes and principal cells of the cortical collecting duct (CCD), was tested. In podocytes, a rise in extracellular zinc promotes TRPC6 channel-mediated calcium entry but not altered intracellular zinc levels. However, we observe the opposite effect in CCD cells with no alteration in calcium levels and steady-state elevation in intracellular zinc. Moreover, prolonged extracellular zinc exposure leads to cytotoxic insults in CCD cells but not in podocytes, characterized by increased cell death and disrupted cytoskeletal organization. Next, we tested if dietary zinc plays a role in the development of hypertension in Dahl salt-sensitive rats. Neither zinc-rich nor deficient diets impact the regular development of salt-sensitive hypertension. These results suggest specialized roles for zinc in renal function, implicating its involvement in proliferation and apoptosis in CCD cells and calcium signaling and cytoskeletal dynamics modulation in podocytes. Further research is required to elucidate the detailed mechanisms of zinc action and its implications in renal health and disease.

Zinc plays multiple roles in cell signaling, acting as a critical mediator in various physiological and biochemical processes. As an essential trace element, zinc stabilizes protein structures and modulates the activity of enzymes, ion channels, and receptors, highlighting its importance in intracellular and intercellular processes. Additionally, it serves as a signaling metal ion alongside Ca^2+^ and Mg^2+^, regulating biological processes and acting as a cofactor in numerous zinc metalloproteins (1, 2). Zinc homeostasis is primarily mediated by zinc transporters located on the apical and basolateral membranes of enterocytes, accounting for approximately 80% to 95% of zinc excretion through fecal matter (3, 4). Despite this predominant route, the role of the kidneys in zinc excretion, though smaller in scale, is crucial (5). Only about 2% to 10% of total zinc excretion occurs *via* urine (6, 7). Nevertheless, the renal pathway plays an essential role in maintaining the body’s overall balance of zinc, and more research is needed in this area. Previous studies show that zinc can affect kidney cell function. Zinc has been identified as a crucial element in inhibiting the proliferation of renal cell carcinoma cells, where it induces oxidative stress and mitochondrial injury, leading to autophagy (8). Its protective role is further highlighted in the context of contrast media-induced cytotoxicity, where zinc preconditioning significantly reduces oxidative stress and enhances renal cell survival (9). The impact of zinc extends to the prevention of epithelial-to-mesenchymal transition in renal tubular epithelial cells, a critical process in the development of renal fibrosis and diabetic nephropathy, by inhibiting high glucose-induced oxidative stress and the activation of key signaling pathways (10). As a toxic effect, zinc can induce damage to kidney cells *via* a mechanism reliant on the entry of zinc ions into the cell, their binding to cellular organelles, and the subsequent disruption of cellular functions (11).

Studying the role of zinc in cellular and subcellular functions in the kidney is important since alterations in zinc homeostasis are frequently observed in patients with chronic kidney disease (CKD) (12). Patients with CKD exhibit lower circulating zinc levels and higher urinary zinc excretion compared to individuals without CKD, indicating renal involvement in the development of zinc deficiency (13). Since zinc is involved in various cellular processes and signaling, elevation in zinc urinary levels could trigger different cell responses along the nephron. Additionally, recent experimental findings suggested a role for zinc in renal damage and its potential contribution to blood pressure regulation, mainly through modulating renal sodium transport (14). Zinc deficiency has been associated with increased blood pressure and an increased risk of hypertension, while zinc supplementation has been shown to exert blood pressure-lowering effects in some experimental models and human studies (13, 15). Zinc deficiency is also often observed in patients with diabetes, and it was proposed that Zn^2+^ intracellular complexes protect the kidneys against diabetes-induced oxidative damage, inflammation, and fibrosis (16, 17). However, the direct mechanism by which zinc deficiency or supplementation affects kidney function remains to be fully understood.

Considering the limited research on the effect of zinc on renal cells and its potential role in modulating blood pressure, we conducted both *in vitro* and *in vivo* experiments to investigate the role of zinc in the kidney. In this article, we explored the impact of extracellular Zn^2+^ on renal epithelial cells and zinc dietary balance on blood pressure. We conducted experiments to observe calcium and zinc intracellular changes in response to extracellular Zn^2+^ elevation in human podocytes and mouse cortical collecting duct (mCCD) cultures. Our findings reveal that extracellular Zn^2+^ has opposite effects on these renal epithelial cells. In podocytes, extracellular Zn^2+^ promotes calcium entry through the transient receptor potential cation channel, subfamily C, member 6 (TRPC6) channels but does not affect zinc levels. In contrast, elevation in extracellular Zn^2+^ produces a rise in intracellular Zn^2+^ in mCCD cells without altering calcium levels. Additionally, we studied the cytotoxic effects of Zn^2+^ and its influence on the cytoskeleton. Prolonged extracellular Zn^2+^ exposure induced mCCD cell death but did not impact podocytes, demonstrating resistance to zinc toxicity. We also found that zinc disrupts the cytoskeleton in mCCD cells, affecting cell structure. Despite these cellular effects, our dietary study in Dahl salt-sensitive (SS) rats showed that neither zinc supplementation nor deficiency significantly altered blood pressure, suggesting that the influence of zinc on blood pressure is limited under the high-salt challenge.

## Results

### Changes in extracellular Zn^2+^ induce calcium entry in podocytes and intracellular zinc elevation in mCCD cells but not *vice versa*

**Figure 1:**
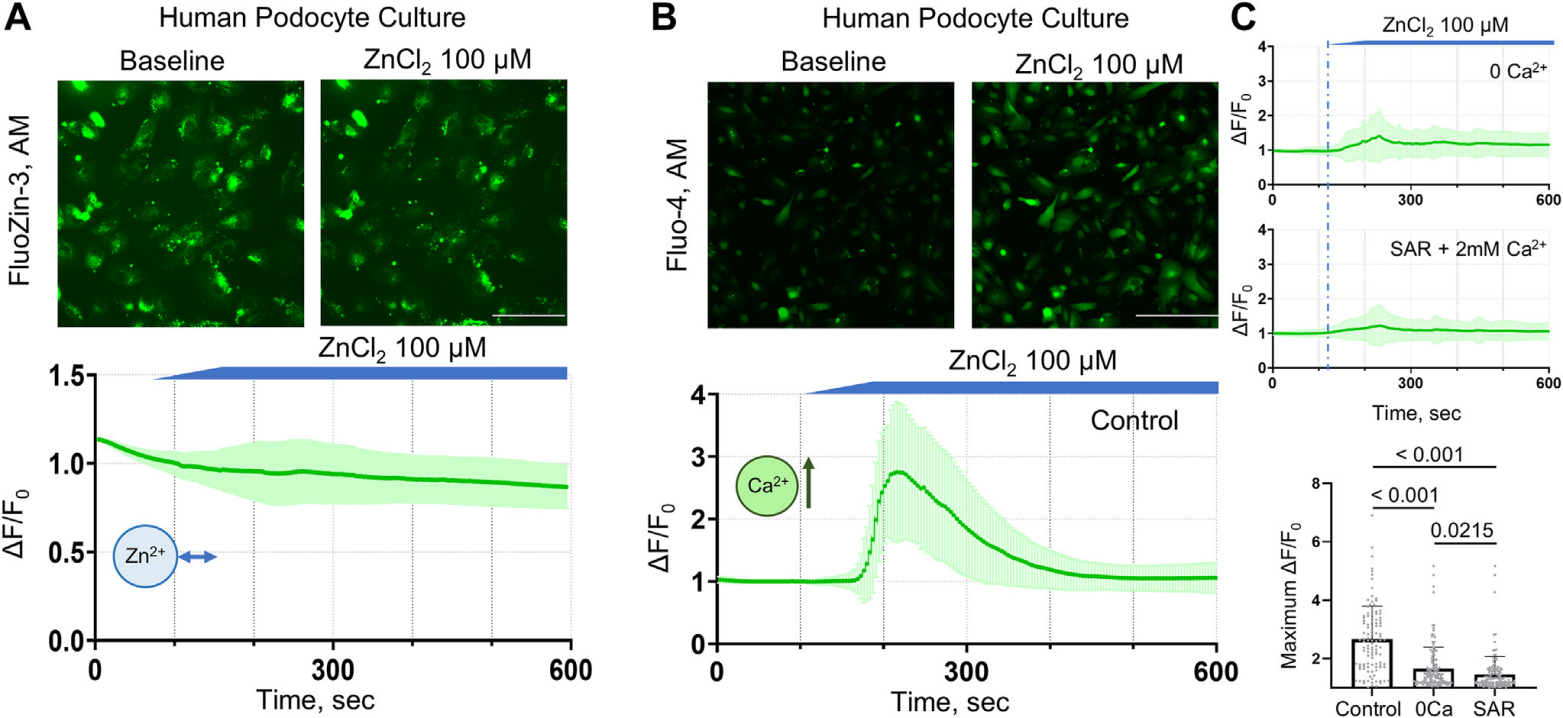
**Zinc induces changes in intracellular calcium levels in podocytes without affecting intracellular zinc levels.** Representative fluorescence images of intracellular zinc (*A*) and calcium (*B*) changes in human podocytes in response to acute application of ZnCl_2_ (100 μM). In podocytes, ZnCl_2_ application did not cause changes in intracellular zinc levels (*A*), but caused changes in intracellular calcium levels (*B*). The statistical summaries show the changes in intracellular calcium with time and maximum ΔF/F_base_ ratio before and after application (podocytes control: 1.2 ± 0.4 vs. 2.6 ± 1.1, n ≥ 20, N ≥ 3, Mann-Whitney, *p* < 0.0001). Additionally, calcium response in podocytes was blunted in 0 Ca^2+^ solution as well as in the presence of SAR7334 (20 μM), a specific TRPC6 inhibitor (*C*; Control vs. 0 Ca^2+^: 2.6 ± 1.1 vs. 1.6 ± 0.7, *p* < 0.0001; Control vs. SAR: 2.6 ± 1.1 vs. 1.4 ± 0.6, *p* < 0.0001; and 0 Ca^2+^ vs. SAR: 1.6 ± 0.7 vs. 1.4 ± 0.6, *p* = 0.0215; n ≥ 20 cells, N ≥ 3, ANOVA). The scale bar is 100 μm.

To uncover the potential mechanism of zinc in the kidney, we tested the effects of extracellular zinc in glomeruli epithelial cells, or podocytes, and the principal cells of the cortical collecting duct (CCD). First, we tested whether increasing extracellular zinc can alter zinc or calcium homeostasis in human podocytes. Application of ZnCl_2_ in a physiological salt solution with Ca^2+^ had no distinctive alterations in intracellular zinc concentrations (Fig. 1*A*). However, we found that there was a significant increase in intracellular calcium concentration [Ca^2+^]_i_ (Fig. 1*B*). Elevation of the [Ca^2+^]_i_ response to ZnCl_2_ was blunted in a 0 mM Ca^2+^ solution, demonstrating that plasma membrane channels are responsible for the Ca^2+^ influx. Members of the TRP family, including TRPC6 channels, are essential mediators of intracellular calcium signaling in podocytes (18, 19). It was also reported that zinc can augment the activity of TRP channels and trigger calcium flux through these channels (20, 21). Therefore, we tested if TRPC6 contributed to the elevated [Ca^2+^]_i_ in response to zinc application. The calcium response was almost completely inhibited in the presence of SAR7334, a specific TRPC6 inhibitor confirming the involvement of TRPC6 channels in zinc-mediated calcium entry in podocytes (Fig. 1*C*).

**Figure 2:**
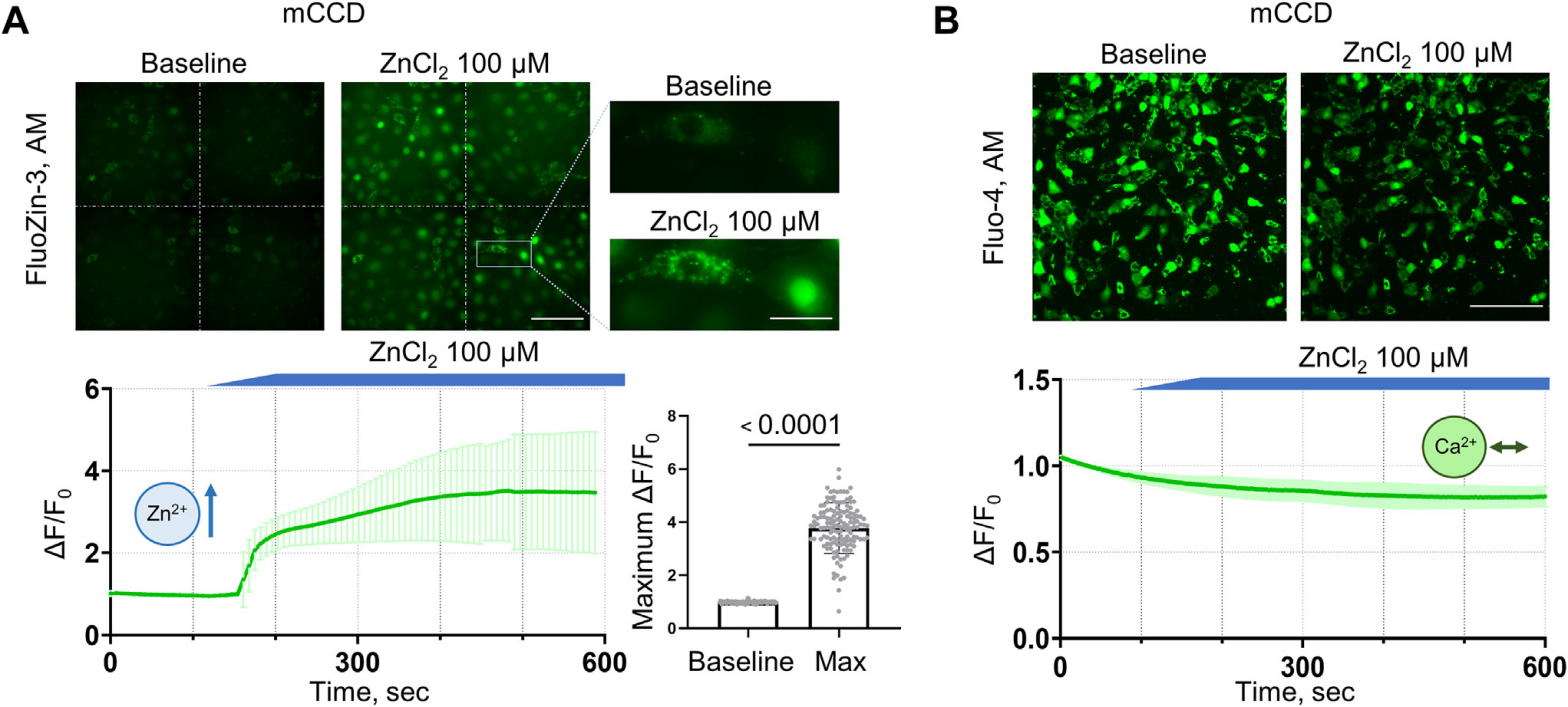
**Zinc induces changes in intracellular zinc levels in mCCD without affecting intracellular calcium levels.** Representative fluorescence images of intracellular zinc (*A*) and calcium (*B*) changes in mCCD in response to acute application of ZnCl_2_ (100 μM). In mCCD, ZnCl_2_ application caused changes in intracellular zinc levels (*A*) but did not cause changes in intracellular calcium levels (*B*). The statistical summaries show changes in zinc with time and maximum ΔF/F_base_ ratio before and after application (mCCD: 0.98 ± 0.03 vs. 3.78 ± 0.95; n ≥ 20 cells, N ≥ 3, *t* test, *p* < 0.001). The scale bar is 100 μm for the main images and 30 μm for the zoomed-in sections.

Similar experiments were done with cultured immortalized mCCD cells. In contrast to podocytes, we found that the application of ZnCl_2_ in mCCD cells triggered a noticeable change in intracellular zinc levels (Fig. 2*A*) but did not elicit a significant increase in [Ca^2+^]_i_ concentrations (Fig. 2*B*), suggesting a divergence in zinc handling or permeability between the two cell types. Interestingly, we observed differential responses to extracellular zinc within the mCCD cell population. We noted a subset of cells exhibited a gradual, uniform increase in intracellular levels of zinc, whereas other cells displayed a scattered pattern of fluorescence, indicating rapid accumulation of zinc in cell organelles, like mitochondria (Fig. 2*A*, *right panel*).

### Zinc induces cell death in mCCD but not in podocytes

**Figure 3:**
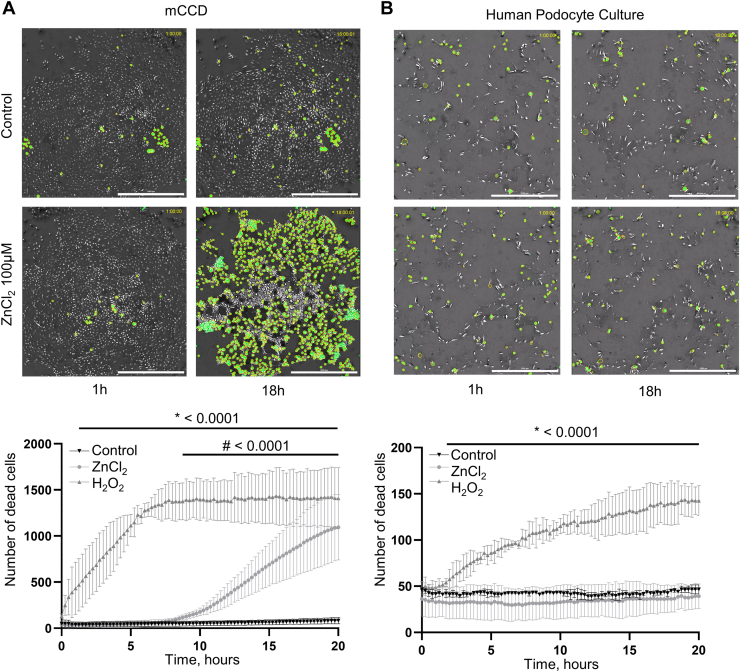
**Zinc induces cell death in mCCD, but not in podocytes.** Representative images of cell death in mCCD (*A*) and immortalized human podocytes (*B*) within 20 h after ZnCl_2_ application. Incubation of podocytes with 100 μM of ZnCl_2_ (∗∗∗, ###*p* < 0.001, ANOVA, N = 6) resulted in a higher number of Nuclear Green-marked cells after 20 h compared to the control. H_2_O_2_ (1 mM) was used as a positive control in both cases. The scale bar is 1 mm.

Previous research has shown that calcium overload can cause podocyte damage through the upregulation of TRPC6 channels (22, 23, 24), and zinc influx can regulate apoptosis and cytotoxic cell death (25, 26). The cytotoxic effects of increased extracellular zinc concentration were further evaluated in podocytes and mCCD within 20 h of cell exposure to 100 μM of ZnCl_2_. H_2_O_2_ is known to promote apoptotic events in podocytes (27). We used a high concentration of H_2_O_2_ (1 mM) as a positive control. Application of H_2_O_2_ resulted in a significant increase in Nuclear Green-positive cells right after application in both cell types (Video S1). In mCCD cells, prolonged incubation in extracellular zinc also promotes cell death (Fig. 3*A*), evident from the increasing number of the Nuclear Green-positive cells starting approximately 7 h post-application (Fig. 3*A*). The extent of cell death in this context was pronounced, signifying the sensitivity of mCCD cells to zinc-induced cytotoxicity. Conversely, podocytes maintained structural integrity and showed resistance to the cytotoxic effects in similar conditions (Fig. 3*B*). These findings underscore a distinct cell-specific response to zinc, with mCCD cells being more vulnerable to zinc-induced cytotoxicity than podocytes.

### Zinc modulates cytoskeleton rearrangements

**Figure 4:**
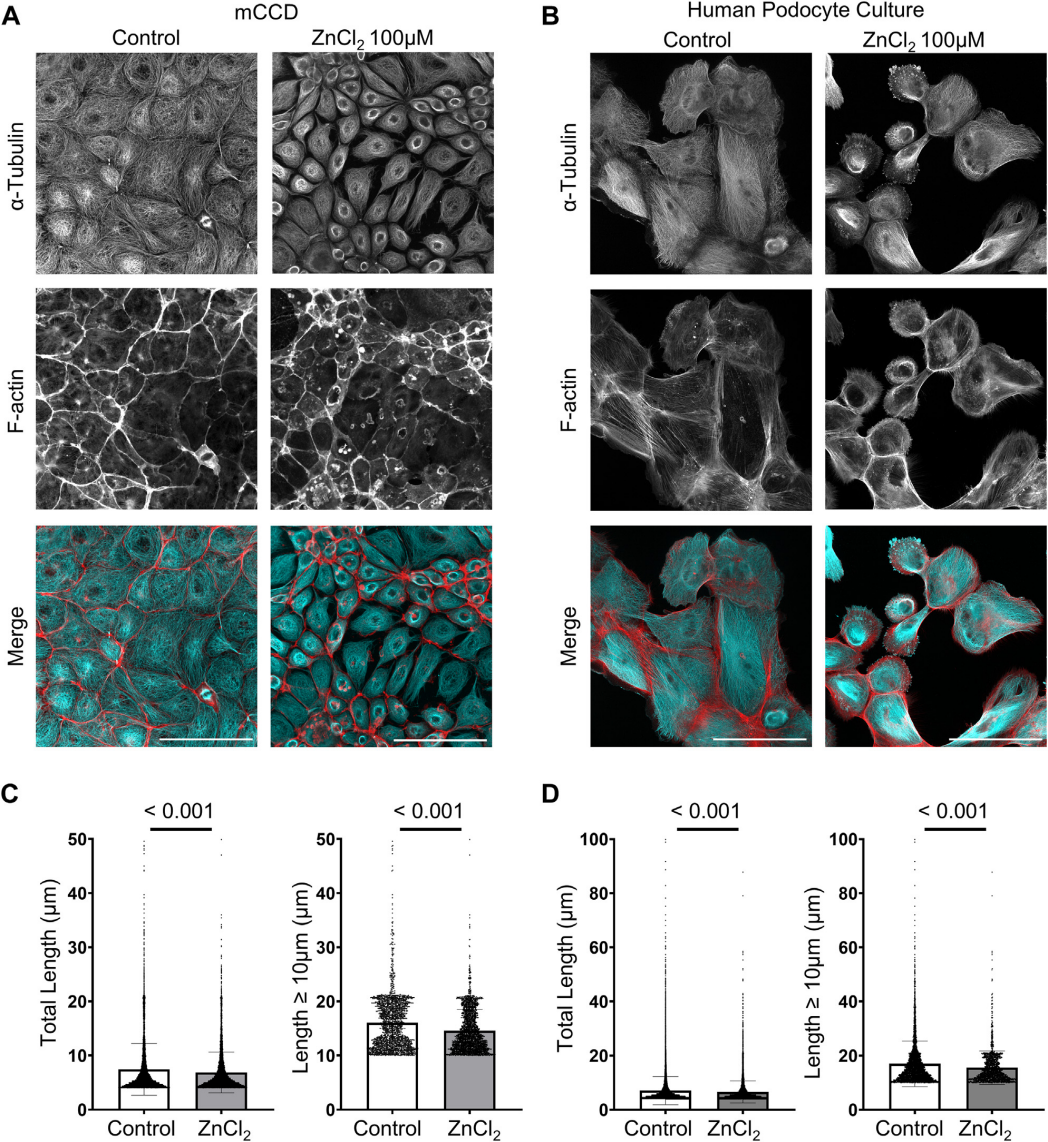
**Impact of zinc on cytoskeletal organization in mCCD cells and podocytes.** Immunofluorescence images of mCCD cells (*A*) and podocytes (*B*) stained for microtubule (α-Tubulin, top row) and actin network (F-actin, middle row) under control conditions and after treatment with ZnCl_2_ (100 μM). In the control group, the microtubule network appears dense and uniformly distributed with fewer gaps and less defined cell boundaries, while the actin filaments form a continuous and intricate network. Upon ZnCl_2_ treatment, the microtubule network becomes more perinuclear dense with more defined cell boundaries, and the actin network shows noticeable fragmentation and reduced filamentous length. In the merged images, cyan represents microtubules (α-Tubulin), and red represents actin (F-actin). Quantitative analysis of the total filament length and the length of filaments ≥10 μm showed a significant reduction in filament length upon ZnCl_2_ treatment compared to controls, both in mCCD cells (*C*, Total length: Control 7.4 ± 4.8 vs. Zn 6.9 ± 3.8 μm; Length ≥ 10 μm: Control 16 ± 5.2 vs. Zn 14.6 ± 3.9 μm; n ≥ 3, N ≥ 3, Kolmogorov-Smirnov, *p* < 0.001) and in podocytes (*D*, Total length: Control 7 ± 5.2 vs. Zn 6.6 ± 4 μm; Length ≥ 10 μm: Control 16 ± 8.4 vs. Zn 15.6 ± 6.2 μm; n ≥ 3, N ≥ 3, Kolmogorov-Smirnov, *p* < 0.001). The scale bar is 100 μm.

Calcium influx and the activation of TRPC6 channels can initiate various downstream signaling pathways that are crucial for cytoskeletal reorganization (28, 29). Similarly, the influx of zinc into cells significantly alters cytoskeletal architecture by interacting with and modulating key cytoskeletal components, thereby impacting cell structure, motility, and signaling pathways (30, 31). Our investigation into the effects of zinc on cellular architecture revealed that zinc exposure induces substantial cytoskeletal rearrangements in renal epithelial cells. Specifically, mCCD cells and podocytes treated with ZnCl_2_ showed pronounced changes in cytoskeletal organization compared to control cells (Fig. 4, *A* and *B*).

In the control mCCD and podocyte, the microtubule network formed a notably dense and tightly packed structure with a relatively uniform and compact pattern, displaying fewer gaps and less distinct cell boundaries (Fig. 4, *A* and *B*; Control, α-Tubulin). Actin filaments also exhibited a well-organized structure, forming an intricate and continuous network (Fig. 4, *A* and *B*; Control, F-actin), indicative of a stable and intact renal epithelial cell organization.

Exposure to ZnCl_2_ led to significant alterations in both microtubules and actin structural patterns. In mCCD cells, the microtubule network became less dispersed, accompanied by the formation of actin stress fibers and the assembly of focal adhesions. This reorganization resulted in a less compact overall structure, with visible gaps between adherent cells, indicative of disrupted cell-cell adhesion (Fig. 4*A*, ZnCl_2_). Similarly, in podocytes, ZnCl_2_ exposure led to comparable structural alterations, including noticeable cell shrinkage (Fig. 4*B*, ZnCl_2_). This shrinkage suggests the retraction of foot processes, a condition often associated with a loss of podocyte function in maintaining the integrity of the glomerular filtration barrier. These alterations may be attributed to zinc’s ability to bind to and modulate the function of key cytoskeletal proteins, thereby affecting cell shape, mechanical properties, and intracellular transport dynamics.

### Zinc supplements or zinc-deficient diets do not affect the progression of salt-induced hypertension in Dahl SS rats

**Figure 5:**
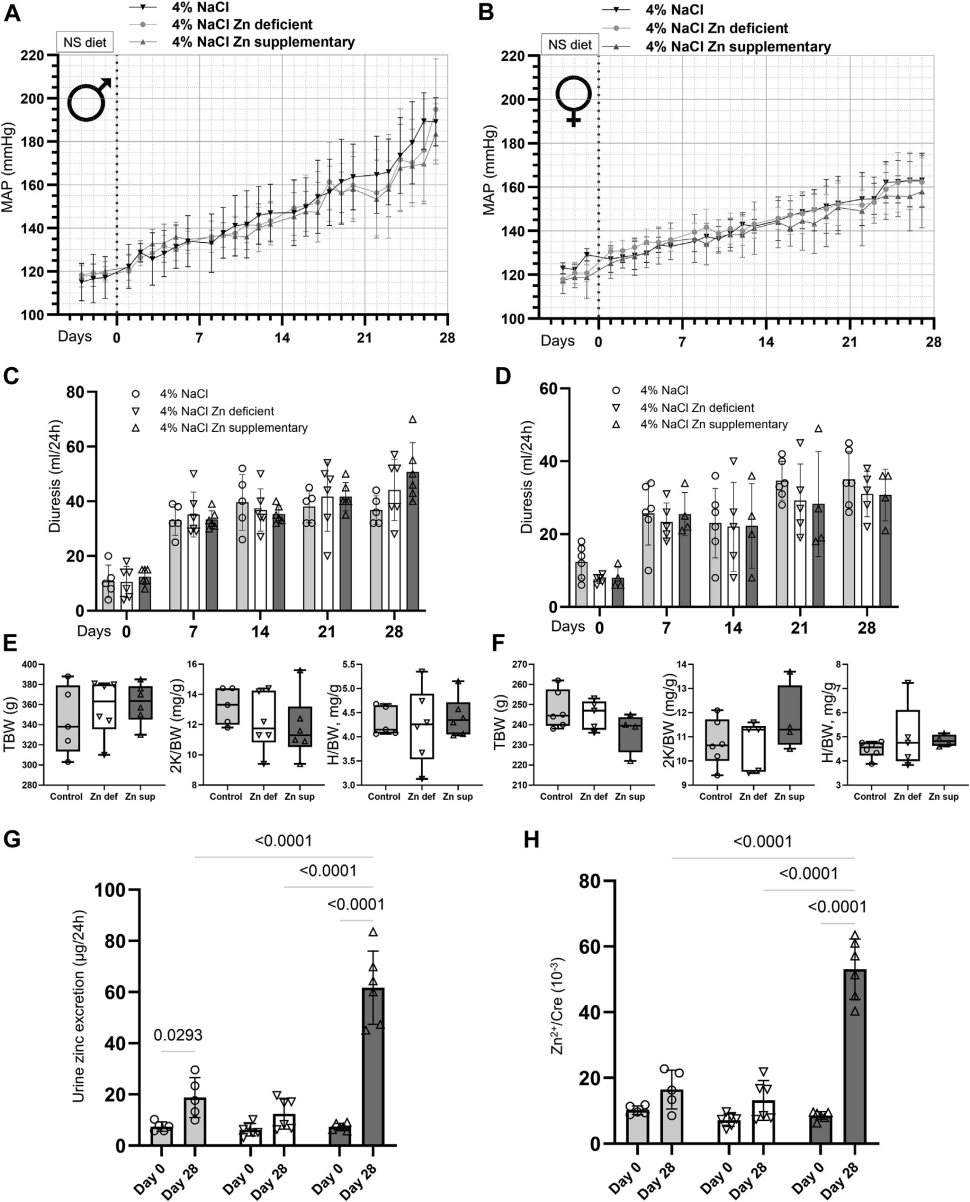
**Zinc supplementary or zinc-deficient diets do not affect salt-induced hypertension in Dahl salt-sensitive (ss) rats.** The development of mean MAP in male (*A*) and female (*B*) Dahl SS rats fed with zinc-deficient (<5 ppm) or zinc-supplementary (180 ppm) high-salt diets over 28 days. We observed no difference in MAP between the supplementary and deficient diets. Additionally, no changes were detected in diuresis (*C*, *D*), urine electrolytes, kidney weight, heart weight, and body weight (*E*, *F*) under zinc-deficient or zinc-supplementary high-salt diets within 28 days. These results suggest that zinc supplementation does not have a significant impact on blood pressure and renal function in Dahl SS rat model of salt-sensitive hypertension. 24 h zinc excretion (*G*), as well as zinc/creatinine ratio (*H*), were significantly higher in the zinc-supplementary group at Day 28, compared to control and zinc-deficient groups. *Light grey*, *white*, and *dark grey* represent control, zinc-deficient, or zinc-supplementary diets, respectively.

Based on growing evidence that zinc supplementation may reduce blood pressure (32) and zinc deficiency can cause the opposite effect (14), we decided to test this hypothesis *in vivo*. The influence of dietary zinc on salt-induced hypertension was assessed in Dahl SS rats, a recognized model for studying SS hypertension. The impact of dietary zinc restriction and supplementation was tested similarly to the protocols described recently (33). Over a 28-day period, both male (Fig. 5*A*) and female (Fig. 5*B*) SS rats were subjected to either zinc-deficient (<5 ppm) or zinc-supplementary (180 ppm) diets, concurrently with a high-salt challenge (4% NaCl). The mean arterial blood pressure (MAP) measurements did not show any significant differences attributable to zinc intake in both genders. Further parameters, including kidney, heart, and body weights (Fig. 5, *E* and *F*), remained unchanged, as well as diuresis (Fig. 5, *C* and *D*) and urine electrolyte composition (Table 1) under varying zinc intake in both genders. To confirm the effectiveness of manipulation with zinc concentration in the diet, we assessed zinc excretion. Urine zinc excretion was measured in male groups on Day 0, before the diet change, and on Day 28, after the diet change. 24-h zinc excretion, as well as zinc/creatinine ratio, were significantly higher in the zinc-supplementary group at Day 28 compared to control and zinc-deficient groups. Interestingly, in the control group, there was a significant increase in 24-h zinc excretion between Day 0 and Day 28. The absence of any discernible effect supports the conclusion that in the context of a high-salt diet, zinc availability does not modulate hypertension development or contribute to renal hypertrophy within the timeframe of this study.

**Table 1:** Urine electrolyte composition in Dahl SS rats fed normal (Day 0) and high salt diets (Day 28) with various zinc concentrations

Urine electrolytes	Day 0			Day 28		
	Control	Zn def	Zn sup	Control	Zn def	Zn sup
Males						
*K*^*+*^*/Cre*	13.3 ± 0.9	12.7 ± 1.6	12.3 ± 1.5	12.6 ± 0.4	12.7 ± 1.6	11.5 ± 1.7
*Na*^*+*^*/Cre*	10.1 ± 1.8	10.6 ± 1.9	12.6 ± 0.4	109.7 ± 5.8	90.6 ± 22.5	91.9 ± 17.5
*Ca*^*2+*^*/Cre*	0.1 ± 0.03	0.05 ± 0.01	0.09 ± 0.04	0.7 ± 0.1	0.7 ± 0.2	0.6 ± 0.2
*Cl*^*-*^*/Cre*	19.9 ± 3.5	16.9 ± 3.8	19.1 ± 4.2	107.7 ± 5.6	95.3 ± 22.1	95.6 ± 0.4
Females						
*K*^*+*^*/Cre*	18.5 ± 12	17.1 ± 2	14.6 ± 2.6	14.8 ± 2.8	16.3 ± 2	18.8 ± 3.6
*Na*^*+*^*/Cre*	11.8 ± 6.4	14 ± 2.1	10 ± 2.3	112 ± 31	126.2 ± 25.1	141.9 ± 25.8
*Ca*^*2+*^*/Cre*	0.3 ± 0.1	0.3 ± 0.1	0.2 ± 0.1	1 ± 0.4	1.6 ± 0.9	1.4 ± 0.3
*Cl*^*-*^*/Cre*	14.6 ± 3.7	22.4 ± 3.7	16 ± 4.2	154.5 ± 28.9	126.5 ± 22.8	139.6 ± 11.4

## Discussion

This study explores the potential cellular responses to extracellular zinc in renal epithelial cells and the implications of dietary zinc content on the development of hypertension. The role of zinc as a modulator of intracellular ion levels in renal cells was investigated through its effect in cultures of the mCCD principal cells and human podocytes. Our findings highlight a differential cellular response to zinc exposure, providing insight into the cell-specific ionic homeostasis within the renal system. The data revealed that zinc modulates [Ca^2+^]_i_ levels in podocytes primarily through TRPC6 channels, a finding that underscores the intricate relationship between zinc and calcium signaling in renal cells. The interplay of intracellular calcium and zinc ions is essential for various cellular processes, including neurotransmission, cell signaling, and metabolism. Zinc can influence calcium dynamics by modulating the activity of calcium channels and receptors. For example, zinc can modulate the activity of voltage-dependent calcium channels (VDCCs), N-methyl-D-aspartate receptors (NMDA), and amino-3-hydroxy-5-methyl-4-isoxazolepropionate receptors (AMPA), which are all calcium-permeable channels, with AMPA receptor permeability for Ca^2^⁺ depending on the receptor composition (34, 35). Notably, NMDA receptors are widely expressed in the kidney, particularly in podocytes (19, 36, 37, 38). Interestingly, despite the fact that TRPC6 channels are permeable to zinc ions, we did not observe an increase in intracellular zinc concentration in podocytes. The presence of NMDA receptors in podocytes suggests a potential role in the zinc-mediated calcium response. This hypothesis and the observed lack of zinc influx in podocytes require additional study.

Conversely, in mCCD cells, the presence of extracellular zinc increased cytosolic zinc concentrations without altering intracellular calcium levels. Additionally, as mentioned in the results, we observed differential responses to zinc within mCCD cells. These distinctive differences in zinc response might be indicative of varied zinc handling and storage mechanisms in mCCD cells. It is well-established that zinc homeostasis is tightly regulated within cells, encompassing a sophisticated network of zinc transporters such as ZnT and ZIP families (39). Indeed, the observed whole-cell fluorescence could be reflective of a global increase in cytosolic zinc levels, potentially mediated by the upregulation of zinc influx transporters such as ZIP14 (40). On the other hand, the 'vesicle-like' zinc deposition might suggest a more localized zinc handling mechanism, possibly related to zinc storage in the endoplasmic reticulum, Golgi apparatus, or mitochondria, which are known to harbor high zinc concentrations (41). Intriguingly, the ZnT family of transporters, particularly ZnT6, has been implicated in the sequestration of zinc into intracellular vesicles, a mechanism that could potentially explain our observed vesicle-like fluorescence (42). The involvement of ZIP and ZnT transporters in zinc signaling in mCCD cells needs further investigation.

Free zinc ions outside a narrow concentration range can be toxic to a variety of cells, including PC-12, HeLa, HT-29 cell lines, as well as primary cultures of cardiac myocytes and neurons (43). Zinc toxicity can induce various signaling cascades, including the activation of protein kinase C, extracellular signal-regulated kinases 1/2, and neuronal nitric oxide synthase, leading to the production of reactive oxygen species and nitric oxide, which can ultimately cause cell death. Conversely, in the context of kidney cells, some studies reported that extracellular Zn^2+^ at concentrations around 100 μM could have protective effects (44, 45). For example, this concentration was most effective in protecting human kidney proximal tubular cells from depleted uranium-induced apoptosis, as well as 50 μM modulates high glucose-induced apoptosis by suppressing oxidative stress in renal tubular epithelial cells. In our experiments, the response to excess zinc varies significantly between mCCD cells and podocytes. The mCCD cells, which exhibit pronounced cytotoxic effects from increased extracellular Zn^2+^, highlight a vulnerability that could stem from less effective protective mechanisms against zinc toxicity. Interestingly, despite the potential role of calcium influx in inducing cell death in podocytes through the upregulation of TRPC6 channels (27, 46, 47), podocytes did not exhibit cytotoxic effects from increased extracellular Zn^2+^ and zinc-mediated calcium influx. This suggests potential protective mechanisms that effectively counteract the toxic effects of zinc, contrasting with the sensitivity observed in mCCD cells. These mechanisms in some kidney cells include the induction of metallothionein and zinc transporter 1 (ZnT1), which bind and sequester free zinc ions (48). Further research could elucidate these mechanisms.

Moreover, zinc not only affects cell viability but also influences cellular architecture, particularly the cytoskeleton. The influx of calcium and the activation of TRPC6 channels can induce changes in the cytoskeletal structure through calcium-dependent signaling pathways (28, 46, 49), which are vital for cell survival. In our observations, cytoskeletal reorganization occurred in podocytes, likely prompted by calcium influx; however, this did not affect cell viability during the period of our experiment. It should be noted that the experiments were conducted using different solutions, specifically culture media *versus* PSS. The binding nature of zinc in culture media may have affected the free zinc concentration, potentially leading to a reduced toxic effect in the cytotoxicity assay. Similarly, the influx of zinc into mCCD cells leads to substantial cytoskeletal changes, potentially impacting essential cellular components and influencing cell structure and viability through unknown signaling pathways. This disruption suggests that zinc may interact directly with actin or actin-binding proteins, disrupting the actin polymerization process and the stability of the cytoskeletal network. Exploring these mechanisms could be a valuable direction for future research.

Furthermore, our study extended to assess the impact of dietary zinc on the development of blood pressure in Dahl SS rats, an established model of salt-induced hypertension. Our results showed no significant differences in blood pressure, renal function, or body weight under varying zinc intake conditions. The effect of zinc on blood pressure in the context of kidney function is complex, with studies indicating both protective and detrimental roles depending on zinc levels and the physiological state of the kidney. Hypertension in Dahl rats led to decreased copper levels in the liver, kidney, and heart, increased plasma copper, elevated tissue zinc, and reduced plasma zinc, indicating altered copper and zinc metabolism (50). Excessive zinc intake has been shown to increase MAP and kidney angiotensin II levels while reducing renal blood flow and inulin clearance, suggesting a reduction in renal function (51). This deterioration in renal function due to zinc excess is further supported by findings that elevated zinc intake leads to increased systemic blood pressure and reduced renal blood flow through mechanisms involving oxidative stress (52). Conversely, zinc deficiency, a common issue in patients with CKD, is associated with hypertension and exacerbates the condition (14, 53). Zinc supplementation in patients undergoing hemodialysis, who often suffer from zinc deficiency, has been shown to improve systolic and diastolic blood pressure (54), indicating a potential therapeutic role of zinc in managing hypertension in CKD. The relationship between zinc status and blood pressure regulation involves complex mechanisms. Zinc deficiency has been linked to hypertension by reduced renal Na^+^ excretion and Na^+^-Cl^-^ cotransporter (NCC) upregulation, suggesting that zinc plays a role in modulating blood pressure by influencing renal sodium handling (5, 14). Expression of NADPH oxidase-2, which is elevated in response to zinc deficiency, could contribute to the detrimental effects of this diet by causing increased H_2_O_2_ generation, oxidative stress, and kidney hypertrophy (55). Zinc deficiency in mice increases urinary sodium excretion, suppresses Na^+^/H^+^ exchanger (NHE3) expression in the proximal tubule, and upregulates NCC. Yet, these effects are reversed by combining HS and a zinc-deficient diet, which also raises blood pressure in these mice (56). Moreover, the impact of zinc on blood pressure can be traced back to its influence during critical periods of renal development, with zinc deficiency leading to elevated blood pressure and renal dysfunction in adult life due to morphological and functional alterations in the kidney (57). While excessive zinc intake can impair renal function and elevate blood pressure, zinc deficiency is also detrimental, exacerbating hypertension in CKD patients. Zinc supplementation has shown promise in improving blood pressure control in zinc-deficient individuals, highlighting the importance of maintaining optimal zinc levels for kidney health and blood pressure regulation. Potential reasons that we did not observe the effect of zinc on blood pressure may be due to several factors. For example, some research indicates that zinc deficiency, rather than just low zinc levels, is more strongly associated with increased blood pressure. Moderate to severe zinc deficiency seems to be required to observe the blood pressure-lowering effects of zinc supplementation (58, 59). In our study, the zinc-deficiency group did not have a lower zinc level in urine compared to the control, suggesting normal zinc status. This may be due to the duration of supplementation with diets. Longer durations may be required to see blood pressure-lowering effects compared to shorter supplementation periods. Additionally, the balance and interactions between zinc and other minerals, such as copper and selenium, may also influence the impact of zinc supplementation on blood pressure (60).

This study, while providing initial insights into zinc-induced cytotoxicity in mCCD cells and its absence in podocytes, has several limitations. First, it exclusively uses a single 100 μM zinc concentration, not exploring a broader range of concentrations that might reveal dose-dependent cellular responses. Determining physiologically relevant zinc concentration can be challenging. Under normal physiological conditions, the total plasma zinc concentration typically ranges around 10 μM (61, 62). Intracellular total zinc concentration can reach 100 μM (58, 63). However, the approximate concentration of free zinc in the cytoplasm usually ranges from 10 to 100 pM. Significant concentration variations may exist in some instances, potentially due to environmental variations, such as oxidative conditions, interactions of zinc with other proteins, protein folding, and the techniques utilized for measurement. When zinc acts as a signaling molecule, as in the zinc spark or zinc wave, the cellular Zn^2+^ concentration fluctuates in response to various biological stimuli and can reach the micromolar range (59, 63). Such conditions, which can trigger zinc release from proteins or cytosolic compartments, may occur in the kidney under both normal and pathological conditions. Under normal conditions, cellular zinc levels return to normal concentrations within minutes, but under pathological conditions, increased cellular zinc levels can be sustained, leading to toxicity (44). It's important to note that while 100 μM zinc is higher than typical physiological levels, it still provides valuable mechanistic insights into cellular responses to zinc overload or extreme conditions. Additionally, the study does not account for variations in free zinc concentrations due to its binding nature in solution, which might affect the bioactivity of zinc. A more precise control over zinc concentrations, utilizing accurately buffered Zn^2+^ solutions, could enhance the reliability of future dose–response studies. The implications of zinc toxicity over longer durations or beyond the acute exposure of 20 h were also not assessed, which could limit understanding of the toxic effect of zinc. Furthermore, in our investigations using Dahl SS rats, the study duration or the animal model itself may have restricted our ability to detect the significant effects of zinc on salt-induced hypertension. This suggests that alternative models or extended durations could potentially provide different results. These factors collectively underscore the need for further research to comprehensively delineate the impact of zinc across different contexts and conditions.

In summary, our findings suggest that zinc can initiate a transient elevation in [Ca^2+^]_i_ levels in podocytes and intracellular zinc levels in mCCD cells. These responses may potentially activate specific downstream signaling cascades that are significant in cytotoxicity and cytoskeleton remodeling. Moreover, they might be involved in regulating blood pressure, as reported in other animal models. Our data indicate that podocytes and mCCD cells may possess distinct mechanisms for managing extracellular zinc, which could relate to their unique physiological functions and the role of zinc in these cell types. The physiological implications of these findings imply specialized roles for zinc in kidney function. In podocytes, zinc-induced calcium signaling could affect processes such as cell shape changes and motility, which are crucial for maintaining the filtration barrier. In mCCD cells, the role of zinc might be more related to cellular repair, proliferation, or apoptosis, which is essential for preserving the integrity and function of the collecting duct system and may affect blood pressure regulation. Further investigations into the expression and regulation of zinc transporters, channels, binding proteins, and the downstream signaling pathways involved are necessary to fully understand the observed differences.

## Experimental procedures

### Animals

Animal experiments and procedures adhered to the National Institutes of Health (NIH) Guide for the Care and Use of Laboratory Animals, and the protocols were reviewed and approved by the University of South Florida (USF) Institutional Animal Care and Use Committee. Eight-week-old male Dahl salt-sensitive rats (SS; SS/JrHsdMcwi; RRID: RGD_61499) were fed normal salt (NS) diet (0.4% NaCl, #113755, Diets Inc). For the high salt challenge, rats were placed on a 4% NaCl high salt (HS) diet (D113756, Diets Inc). Rats were maintained on an HS diet for 4 weeks and treated with zinc-deficient (<5 ppm) or zinc-supplemented (180 ppm) high-salt diets. Zinc concentration in the control diet is approximately 30 ppm. Water and food were provided *ad libitum*. We used deionized water to minimize additional zinc intake.

### Surgical procedures

At the age of 8 to 8.5 weeks, rats were anesthetized on a temperature-controlled platform *via* inhalation of 2.5% isoflurane in 0.5 L/min (O_2_/N_2_: 30%/70%). A blood pressure transmitter (HD-S10; Data Sciences International, New Brighton, MN) was implanted subcutaneously. The catheter tip was secured in the abdominal aorta *via* the femoral artery (60, 64). Rats were allowed to recover for 4 to 5 days, and blood pressure and heart rate were recorded using DSI software. At the end of the experimental timeline, rats were anesthetized with 5% isoflurane, and kidneys were flushed with phosphate-buffered saline *via* aortic catheterization (65, 66, 67). The left kidney was snap-frozen, and the right kidney was placed in 10% formalin.

### Electrolyte and zinc concentration measurements

Urine was collected for 24 h in metabolic cages (no. 37000M071, Tecniplast) at baseline and every 7 days of the HS protocol. Prior to euthanasia, blood samples were collected by aortic catheterization in anesthetized animals. Glucose, creatinine, and electrolytes (Na^+^, K^+^, Ca^2+^, Cl^-^) in plasma and urine were measured with a blood gas analyzer (ABL system 800 Flex, Radiometer). Measurement of zinc concentration in end-point urine samples was done by The Texas A&M Veterinary Medical Diagnostic Laboratory (TVMDL) according to the company's protocol using inductively coupled plasma/mass spectrometry (ICP/MS).

### Intracellular calcium and zinc measurements in cultured human podocytes and in mCCD cells

Immortalized human podocyte cell line AB 8/13 was kindly provided by M. Saleem and has been described previously (46, 68). Podocytes were cultured on glass-bottom dishes (Mattek, No.0 coverslip) in an RPMI-1640 (Gibco) medium supplemented with 10% heat-inactivated FBS (Corning), insulin-transferrin-selenium supplement (Gibco), with Penicillin-Streptomycin (Cytiva). Podocytes were taken 12 to 14 days after thermo-switching. mCCD_cl1_ cells (69) were provided by Prof. Bernard Rossier (University of Lausanne, Switzerland) and were on glass-bottom dishes (Mattek, No.0 coverslip) in DMEM (DMEM/F-12, Cat #11330, Gibco) supplemented with 2% FBS (Corning), insulin-transferrin-selenium supplement (Gibco), and Penicillin-Streptomycin (Cytiva). as previously described (69, 70). Then, cells were loaded with Fluo-4, AM (#20551, AAT Bioquest) or FluoZin-3, AM (#F24195, Thermo Fisher Scientific) fluorescent dyes according to manufacture protocol and incubated at 37 ^o^C for 1 h. Cells were rinsed, and media was replaced with bath solution (145 mM NaCl, 4.5 mM KCl, 2 mM CaCl_2_, 2 mM MgCl_2_, 10 mM HEPES, pH=7.35). To block TRPC6 channels in experiments with podocyte, SAR7334 (#5831, Tocris Bioscience) at a concentration of 20 μM was added for 30 min before imaging. ZnCl_2_ (100 μM) was added to the solution to evaluate intracellular calcium and intracellular zinc responses. All experiments were done with continuous perfusion (1.5 ml/min). Records were obtained using the Cellinsight CX7 HCS (Thermo Fisher Scientific) microscope system (Olympus LUCPlanFL N 20 × /NA 0.45) and analyzed using the Fiji image processing package.

### Cytotoxicity assay

Immortalized human podocyte and mCCD cells were cultured in a 96-well dish (Thermo Fisher Scientific). To evaluate zinc-induced cytotoxic effects, the Nuclear Green DCS1 (1 μM, #17550, AAT Bioquest) cell-impermeant dye was added in cell medium before treatment with vehicle, ZnCl_2_ (100 μM) or H_2_O_2_ (1 mM). Cells were maintained in culture media, and the treatment was administered directly prior to imaging. The extent of cell death was assessed by quantifying the number of Nuclear Green-positive cells, indicative of compromised cell nuclei. Data were collected using BioTek Cytation C10 (1320516/10× PL FL Phase) in high contrast and epi-fluorescence modes with a montage of four fields of view for 20 h at 37 °C and 5% CO_2_ and analyzed with Gen5 software.

### Immunofluorescence staining for cytoskeletal analysis

To detect cytoskeletal remodeling, cells were incubated in saline solution (see above) with ZnCl_2_ (100 μM) or vehicle for 30 min. After treatment, cells were fixed with 4% paraformaldehyde in PBS, treated with 0.1% Triton X-100 in 2% BSA-PBS, and incubated with α-Tubulin antibody (1:100, #PA5-19489, Thermo Fisher Scientific) for 2h. Next, cells were washed 3× with PBS and incubated with Alexa 488 fluorophore–labeled secondary antibody (1:500, #A-21206, Thermo Fisher Scientific) and rhodamine phalloidin (1:500, #R415, Thermo Fisher Scientific) for 1h. Cells were imaged with the BioTek Cytation C10 (1220545/60× PL FL) in confocal mode. Images were analyzed with the open-source software FilamentSensor (71) to quantify the extent of cytoskeletal fragmentation. Detailed settings can be found in supporting information (Fig. S1). Quantitative data were obtained by analyzing images from at least three independent experiments, with a minimum of three fields of view per experiment.

### Statistics

Data are presented as mean ± standard deviation of the mean (SD). In the box plot graphs, the box represents the mean ± SD. Data were tested for normality (Shapiro-Wilk) and equal variance (Levene's homogeneity test). Statistical analysis consisted of one- or two-way ANOVA, Student's *t*-test or Kolmogorov-Smirnov test (GraphPad Prism 10.0), with a *p*-value of <0.05 considered significant. In addition, when an ANOVA test was significant, *post hoc* Holm-Sidak's or Dunn’s multiple-comparison was performed.

## Data availability

All data used in the study are available in this article. Raw data could be provided by the corresponding author upon request.

## Supporting information

This article contains supporting information.

## Conflict of interest

The authors declare that they have no conflicts of interest with the contents of this article.
