# Supplementary material for: Effects of zinc in podocytes and cortical collecting duct in vitro and Dahl salt-sensitive rats in vivo
Source: J Biol Chem. 2024 Sep 12;300(10):107781. doi: 10.1016/j.jbc.2024.107781 (PMC11736004; doi:10.1016/j.jbc.2024.107781)
Supplement: Supporting information [file mmc2.docx]

**Effects of zinc in podocytes and cortical collecting duct *in vitro* and Dahl Salt-Sensitive rats *in vivo***

Ruslan Bohovyk^1^, Olha Kravtsova^1^, Vladislav Levchenko^1^, Christine A. Klemens^1,2^, Oleg Palygin^3,4^, Alexander Staruschenko^1,2,5,*^

^1^Department of Molecular Pharmacology and Physiology, University of South Florida, Tampa, FL 33602

^2^Hypertension and Kidney Research Center, University of South Florida, Tampa, FL 33602

^3^Department of Medicine, Division of Nephrology, Medical University of South Carolina, Charleston, SC 29425

^4^Department of Regenerative Medicine and Cell Biology, Medical University of South Carolina, Charleston, SC 29425

^5^James A. Haley Veterans' Hospital, Tampa, FL 33612

**Supporting Information Contents Page**

Figure S1 Depiction of cytoskeleton rearrangement and software settings. S-2

Video S1 Legend Zinc induces cell death in mCCD, but not in podocytes S-3

**Figure S1**

**
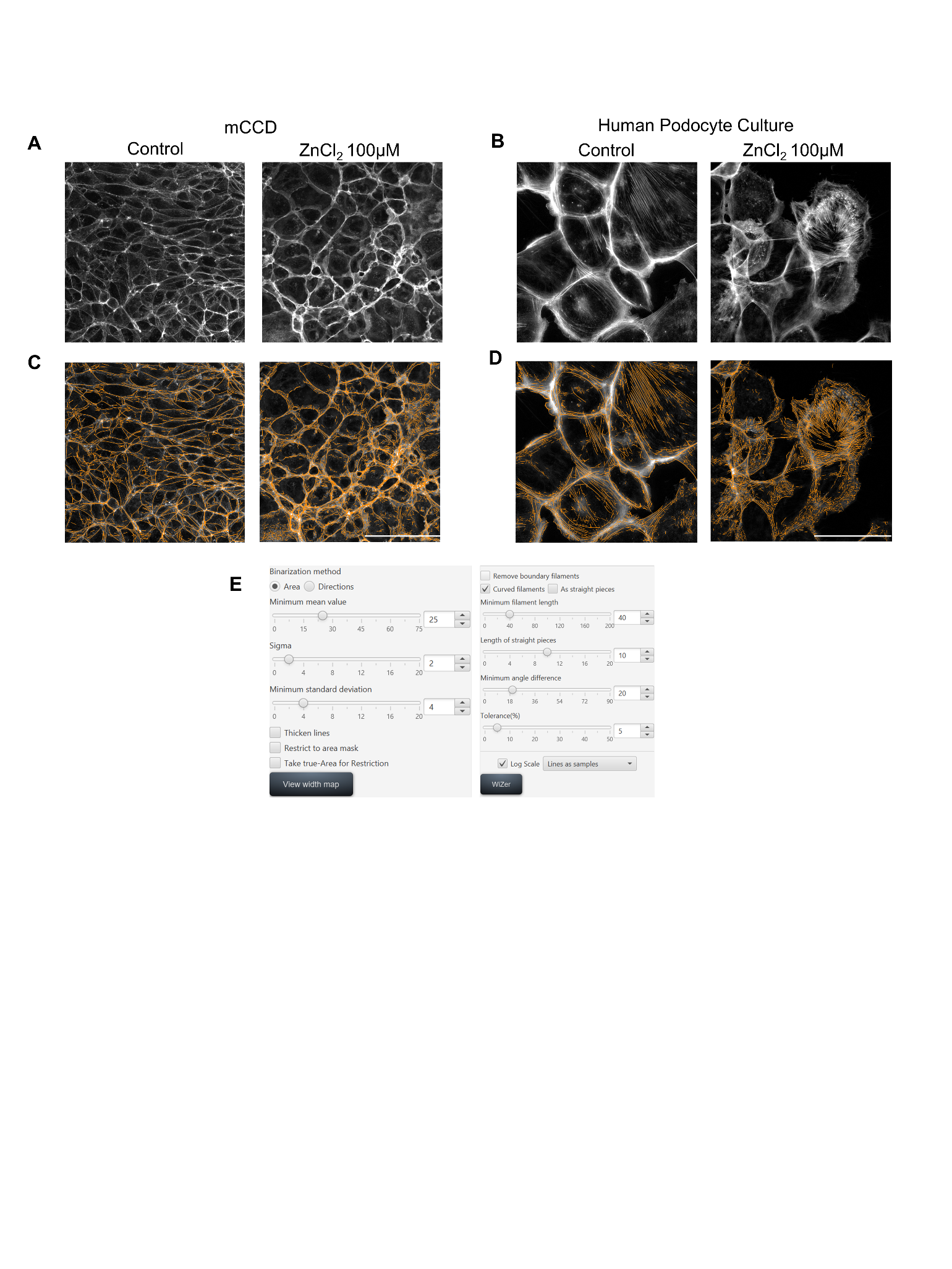
**

Figure S1. Depiction of cytoskeleton rearrangement in mCCD (A) and podocyte cells (B) in response to 30 min exposure to 100 μM ZnCl_2_. Scale bar: 100 μm. Below, images with orange lines represent examples of filament detection in representative frames in mCCD (C) and podocytes (D) groups. (E) Shows the setting of filament sensor software that was used for detection. Images were taken using BioTek Cytation C10 microscope in confocal mode (z-stack, max intensity projection, 60x PL PL, wide field of view camera mode, 231x231 µm, 8.4848 pixels/µm).

**Video S1**

Zinc induces cell death in mCCD, but not in podocytes. Representative video of cell death in mCCD (Top panel) and immortalized human podocytes (Bottom panel) within 20 hrs after ZnCl2 application. Incubation of podocytes with high concentrations of ZnCl2 100 μM (***, ### P < 0.001, ANOVA, N = 6) resulted in a higher number of Nuclear Green marked cells after 20 hrs compared to the control. H2O2 (1 mM) was used as a positive control in both cases. This video represents the changes over time corresponding to the conditions shown in Figure 3. The video frames are derived from the same cell source as those used in Figures 3A and B, demonstrating the dynamic processes observed in the study. The scale bar is 1 mm.
